# The protein kinase SIK downregulates the polarity protein Par3

**DOI:** 10.18632/oncotarget.23788

**Published:** 2017-12-31

**Authors:** Michael Vanlandewijck, Mahsa Shahidi Dadras, Marta Lomnytska, Tanzila Mahzabin, Martin Lee Miller, Christer Busch, Søren Brunak, Carl-Henrik Heldin, Aristidis Moustakas

**Affiliations:** ^1^ Ludwig Institute for Cancer Research, Science for Life Laboratory, Uppsala University, Uppsala, Sweden; ^2^ Department of Medical Biochemistry and Microbiology, Science for Life Laboratory, Uppsala University, Uppsala, Sweden; ^3^ Department of Oncology and Pathology, Karolinska Biomics Center, Karolinska Institute, Stockholm, Sweden; ^4^ Novo Nordisk Foundation Center for Protein Research, University of Copenhagen, Copenhagen, Denmark; ^5^ Department of Immunology, Genetics and Pathology, Rudbeck Laboratory, Uppsala University, Uppsala, Sweden; ^6^ Integrated Cardio Metabolic Center, Novum, Karolinska Institute, Huddinge, Sweden; ^7^ School of Anatomy, Physiology and Human Biology, The University of Western Australia, Crawley, WA, Australia; ^8^ Cancer Research UK, Cambridge Institute, University of Cambridge, Li Ka Shing Center, Cambridge, UK; ^9^ Department of Obstetrics and Gynaecology, Academic Uppsala Hospital, Uppsala, Sweden

**Keywords:** cell junctions, Par3, signal transduction, SIK, TGFβ

## Abstract

The multifunctional cytokine transforming growth factor β (TGFβ) controls homeostasis and disease during embryonic and adult life. TGFβ alters epithelial cell differentiation by inducing epithelial-mesenchymal transition (EMT), which involves downregulation of several cell-cell junctional constituents. Little is understood about the mechanism of tight junction disassembly by TGFβ. We found that one of the newly identified gene targets of TGFβ, encoding the serine/threonine kinase salt-inducible kinase 1 (SIK), controls tight junction dynamics. We provide bioinformatic and biochemical evidence that SIK can potentially phosphorylate the polarity complex protein Par3, an established regulator of tight junction assembly. SIK associates with Par3, and induces degradation of Par3 that can be prevented by proteasomal and lysosomal inhibition or by mutation of Ser885, a putative phosphorylation site on Par3. Functionally, this mechanism impacts on tight junction downregulation. Furthermore, SIK contributes to the loss of epithelial polarity and examination of advanced and invasive human cancers of diverse origin displayed high levels of SIK expression and a corresponding low expression of Par3 protein. High *SIK* mRNA expression also correlates with lower chance for survival in various carcinomas. In specific human breast cancer samples, aneuploidy of tumor cells best correlated with cytoplasmic SIK distribution, and SIK expression correlated with TGFβ/Smad signaling activity and low or undetectable expression of Par3. Our model suggests that SIK can act directly on the polarity protein Par3 to regulate tight junction assembly.

## INTRODUCTION

The transforming growth factor β (TGFβ) pathway is a highly conserved signaling engine [1] that plays major roles in different cellular processes, including proliferation, apoptosis and differentiation [2]. As a consequence, misregulation of TGFβ signaling is involved in several diseases, including cancer [3, 4]. TGFβ shows a prominent role as a tumor suppressor during initial stages of tumorigenesis by preventing cell growth. Nevertheless, it functions as an important promoter of tumor spread in later stages of cancer progression by enhancing tumor cell dedifferentiation towards a more invasive phenotype [5]. TGFβ signaling is initiated by binding of the dimeric TGFβ ligand to a heteromeric receptor complex of two type I (TGFβRI) and two type II (TGFβRII) homodimeric subunits [6, 7]. The kinase of TGFβRII is constitutively active and as a consequence, heteromerization leads to phosphorylation and activation of TGFβRI [8]. This event leads to phosphorylation of the receptor-regulated Smad2 and Smad3, allowing complex formation with the common mediator Smad4. The Smad complexes accumulate in the nucleus where they influence transcription by binding to DNA in cooperation with a plethora of other transcription factors [1, 4, 9].

One of the physiological responses of TGFβ signaling in epithelial cells is their transformation to a more mesenchymal phenotype. This process is called epithelial to mesenchymal transition (EMT) [10]. Hallmarks of EMT include loss of polarity by downregulation of tight junctions (TJ) and adherens junctions (AJ), as well as remodeling of the extracellular matrix and of the cytoskeleton by upregulation of proteins like fibronectin and vimentin [11]. This process normally occurs during embryonic development, but in adults, it has been implicated in pathologic conditions like chronic inflammation leading to tissue fibrosis and cancer invasion [11]. A well-studied mechanism by which TGFβ can induce EMT is through downregulation of the protein E-cadherin, which results from repression of transcription of its gene, leading to breakdown of the AJ [10]. Many transcription factors are known to repress the *E-cadherin* gene*,* including the two-handed zinc finger/homeodomain proteins ZEB1, ZEB2, the basic helix-loop-helix proteins E12/E47, LEF-1 and Twist1, and Snail1 and Snail2/Slug of the Snail family of zinc finger proteins [12]. In recent years, these transcription factors have emerged as major regulators of EMT, repressing more targets besides *E-cadherin,* e.g. *Crumbs,* encoding a regulator of epithelial cell polarity, and *Claudin-1,* encoding a structural member of the TJ [13, 14]. Another mechanism of TJ downregulation involves binding between Par6 and the TGFβ receptor complex, leading to phosphorylation of Par6. This recruits the E3 ubiquitin ligase Smad ubiquitylation regulatory factor 1 (Smurf1) and causes degradation of the small GTPase RhoA, leading to loss of TJ [15]. However, the exact mechanism of TGFβ-induced loss of TJ remains elusive.

The TJ consists of several clusters of transmembrane cell surface proteins and intracellular adaptor molecules. Members of the former group include occludin, claudins, junctional adhesion molecules (JAMs) and Coxsackie-adenoviral receptor (CAR) proteins [16]. All these transmembrane proteins are associated with cytoplasmic regulatory complexes and the actin cytoskeleton via adaptor proteins like the membrane associated guanylate kinase (MAGUK) family that includes the zona occludens (ZO) protein family [17]. These adaptor proteins are an important node for interaction between the TJ and signaling pathways. For example, ZO-1, ZO-2 and cingulin, another important adaptor protein of the TJ network, can regulate the cell cycle, the former by interactions with transcription factors in the nucleus, and the latter by interaction with the oncoprotein guanine exchange factor (GEF)-H1, preventing activation of RhoA [18, 19]. The TJ is also able to interact with an important cluster of the polarity complex, consisting of Par3, Par6 and aPKC (atypical protein kinase C). Par3 binds to JAM-A [20] and Par6 is able to interact with both cell surface molecules (claudins) and adaptor molecules (ZO) through the MAGUK family member PALS1 and the submembrane adaptor PATJ [21]. These interactions allow important crosstalk between the machinery that controls general polarity of the epithelial cells and TJ stability.

Salt-inducible kinase 1 (SIK1, here abbreviated as SIK) was described by our group as a direct target of TGFβ/Smad signaling [22, 23]. SIK is a serine/threonine kinase that belongs to the family of AMP-activated protein kinases (AMPKs) [24]. AMPKs can be phosphorylated by the tumor suppressor LKB1 kinase *in vitro* and thus are thought to contribute to various aspects of LKB1 signaling [25]. SIK expression is induced during cardiogenesis and skeletal muscle differentiation [26], in adrenal glands leading to steroidogenesis [27], in the liver where it suppresses gluconeogenesis [28], and in the brain by the circadian clock transcription factors [29]. SIK consists of an N-terminal kinase domain and a middle ubiquitin-associated (UBA) domain, followed by a long C-terminal sequence with unknown functional motifs [30]. SIK is a highly unstable protein and regulation of its stability contributes to diverse effects ranging from muscle differentiation to kidney physiology [31, 32]. In the context of cancer, SIK has been reported to be required for efficient tumorigenesis in a breast cancer model, and unexpectedly, it was also found to act as a metastasis suppressor by mediating p53-dependent anoikis [33].

We have demonstrated that SIK is a negative regulator of TGFβ signaling because SIK induces downregulation of the TGFβ type I receptor (TGFβRI) [23, 34]. In support of our model, glucose downregulates SIK expression in mesangial cells and causes stabilization of TGFβRI leading to enhanced TGFβ signaling and pro-fibrotic effects [32]. Furthermore, SIK interacts with TGFβ-activated kinase 1-binding protein 2 (TAB2) and interferes with the ubiquitination and activation of the tumor necrosis factor receptor-associated factor 6 (TRAF6), leading to negative regulation of Toll-like receptor signaling in immune cells [35]. Since it is known that TGFβRI is also involved in TJ degradation via its interaction with Par6 [15], we were interested in exploring new functions of SIK in TJ regulation. This paper provides evidence that SIK plays a new role in TJ regulation by degradation of Par3, another key molecule of the polarity complex that is involved in TJ assembly.

## RESULTS

### Novel SIK substrate identification

In order to identify new substrates of SIK with functional relevance to epithelial biology, we performed an unbiased *in silico* screen using all proteins in the SwissProt database and the following short amino acid motif: LxR(S/T)xSxxxL (where x is any amino acid). This motif was derived from the protein sequence of the transcriptional co-activator protein TORC1, formally known as CREB regulated transcription coactivator 1 (CRTC1), which was shown to be phosphorylated directly by SIK [36]. The bioinformatic screen was based on tools developed for the NetPhorest resource [37] and revealed several proteins that contained highly conserved motifs that could serve as putative phosphorylation substrates for SIK (Table 1). Among these proteins, we identified the class II histone deacetylases 4, 5 and 7a (Table 1) that were subsequently shown to be genuine substrates of SIK phosphorylation activity in chemosensory neurons of *C. elegans* by the SIK ortholog KIN-29 [38], in *D. melanogaster* adipose tissue by SIK3 [39], in mouse myotubes by SIK1 [40], in human embryonic cells and mouse myoblasts by SIK2 and SIK3 [41] and in human gastric adenocarcinoma cells by SIK1 [42]. We also identified another modulator of histones, the co-activator protein EP300 (commonly known as p300), which has been previously reported as a substrate of SIK2 (Table 1) [43]. The list of target proteins included additional transcriptional regulators such as the serum-response factor and the retinoblastoma-like protein 2 (p130), signaling proteins such as the serine/threonine kinase Raf1, tuberin (TSC2) and several ion channels (Table 1). Finally, molecules that are known to regulate actin organization (actin-binding LIM protein 1 and cingulin) or proteins that participate in cell-cell junction assembly, such as the polarity protein Par3 [44, 45], scored prominently in this target identification screen (Table 1). The specific sequence identified in Par3 was well conserved among species, with a central serine residue, number 885 in the mouse protein sequence (Supplementary information, Supplementary Figure 1).

**Table 1:** Bioinformatic predictions of SIK substrates

Name	Full name	Uniprot	Target sequence	Ref.
*Cell junction/cytoskeleton*				
PAR3	Partitioning-defective 3 homolog	Q8TEW0	GLKKSSSLESLQT	novel
SIPA1	Signal-induced proliferation-associated 1	O43166	ALHRTLSDESIYN	novel
CTNND2	Catenin-δ2	O35927	TLARSPSIDSIQK	novel
SSH1	Slingshot homolog 1	Q8WYL5	NLTRSSSSDSIHS and PLKRSHSLAKLGS	novel
CGN	Cingulin	Q9P2M7	KLLRSHSQASLAG	novel
ABLIM1	Actin-binding LIM protein 1	O14639	MIHRSTSQGSINS	novel
RACGAP1	Rac GTPase-activating protein 1	Q9P2W2	QLLKTPSSSSLSQ	novel
PSD3	PH and SEC7 domain-containing protein 3	Q80TN6	GLKKSHSSPSLNP	novel
*Ion channels*				
CACNA1S	Voltage-dependent L-type calcium channel subunit α-1S	P07293	FLERTNSLPPVMA	novel
PIEZO1/FAM38A	Piezo-type mechanosensitive ion channel component 1	Q92508	GLMRTASELLLDR	novel
LRRC8C	Volume-regulated anion channel subunit LRRC8C	Q8R502	NLVRSQSLKSIPE	novel
CHRND	Acetylcholine receptor subunit δ	P02718	KLRRSSSVGYISK	novel
*Signal transduction*				
PTH1R	Parathyroid hormone/parathyroid hormone-related peptide receptor	P25107	EIKKSWSRWTLAL	novel
RAF1	RAF proto-oncogene serine/threonine-protein kinase	P04049	KINRSASEPSLHR	novel
GAB1	GRB2-associated-binding protein 1	Q13480	NLPRSYSHDVLPK	novel
TSC2	Tuberin	P49815	PLSKSSSSPELQT	novel
USP8	Ubiquitin carboxyl-terminal hydrolase 8	Q80U87	KLKRSYSSPDITQ	novel
*Metabolism*				
GYS1	Glycogen [starch] synthase, muscle	P13834	PLSRTLSVSSLPG	novel
*Transcription-chromatin remodeling*				
SRF	Serum-response factor	P11831	GLKRSLSEMEIGM	novel
RBL2	Retinoblastoma-like protein 2 (p130)	Q08999	PVMRSSSTLPVPQ	novel
NELFE	Negative elongation factor E	P19426	GVKRSLSEQPVVD	novel
EP300	Histone acetyltransferase p300	Q09472	ELLRSGSSPNLNM	SIK2 [43]
HDAC4	Histone deacetylase 4	P56524	PLRKTASEPNLKL	[38]-[42]
HDAC5	Histone deacetylase 5	Q9UQL6	PLRKTASEPNLKV	[40],[41]
HDAC7A	Histone deacetylase 7A	Q8WUI4	PLRKTVSEPNLKL	[40, 41]

The table lists the official protein name, full name, UniProt number and predicted target sequence that contains putative serine residues where SIK could phosphorylate the target protein. In the last column, novel indicates predicted substrates found for the first time through this work. References to published work indicate identification of substrates that have been previously established. In some cases, the previously established substrates were established for a SIK isoform independent from SIK1, e.g. SIK2, which is noted in the last column. The proteins are divided in functional subgroups.

### SIK associates with Par3

**Figure 1:**
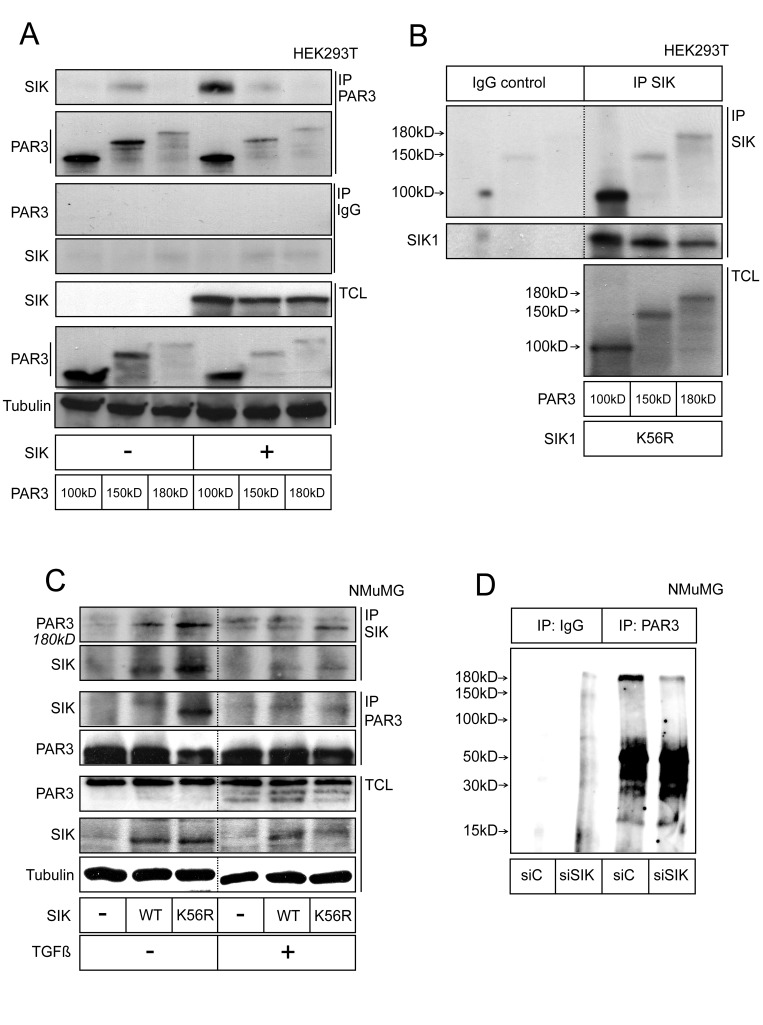
SIK forms a complex with Par3 **A.** Co-immunoprecipitation of SIK with Par3. HEK293T cells were transfected with SIK and the three isoforms of Par3 (termed 180 kD, 150 kD and 100 kD according to their molecular size). Total cell lysates (TCL) are shown, with α-tubulin as loading control. Rabbit IgG was used in control immunoprecipitations. **B.** Co-immunoprecipitation of Par3 with SIK. The same Par3 constructs as in (A) were transfected, together with a kinase-dead mutant of SIK (K56R). An in-house made SIK-specific antibody was used for immunoprecipitation. A non-related antibody was used in control immunoprecipitations. **C.** Semi-endogenous co-immunoprecipitation between transfected SIK and endogenous Par3. Wild-type (WT) and K56R mutant SIK were transfected in NMuMG cells and the immunoprecipitation was performed with either a Par3- or a SIK-specific antibody. The cells were treated with 5 ng/ml TGFβ or vehicle overnight. Dotted lines in panels B and C indicate that intervening lanes of the same membrane have been cut out. **D.** Detection of endogenous phosphorylated forms of Par3. NMuMG cells were transfected with 10 nM of control siRNA or siSIK for 24 h. Afterwards, the growth medium was changed and a second siRNA transfection identical to the first was performed for another 24 h. After lysing, endogenous Par3 was immunoprecipitated and after washing, the beads were incubated with radioactive [^32^P]ATP for 1 h. Following addition of SDS-containing Laemmli buffer and SDS-PAGE, radioactive bands were visualized by autoradiography. The top band corresponds to 180 kD. Representative immunoblots out of at least three repeats are shown in each panel. Molecular size markers in kilo Dalton (kD) are also marked.

**Figure 2:**
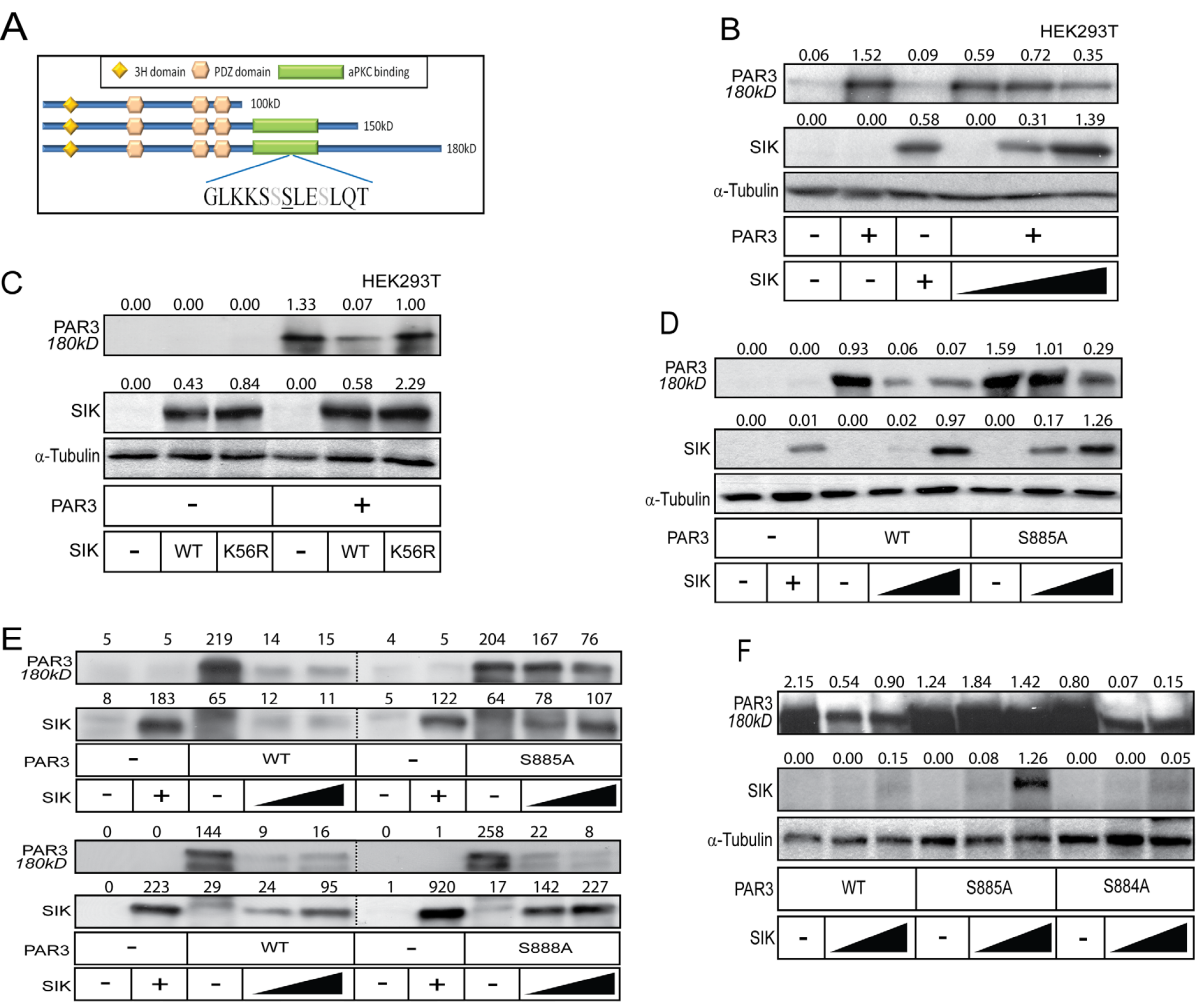
SIK induces degradation of Par3 **A.** Schematic overview of the *in silico* predicted phosphorylation site in Par3. The underlined serine corresponds to Ser885, while the grey serines correspond to Ser884 and Ser888. The Par3 domain organization is also shown. **B.**-**C.** SIK induces degradation of Par3. Increasing amounts of SIK plasmid were co-transfected with full-length Par3 (B) or a single amount of SIK WT and K56R mutant was co-transfected with full length Par3 in HEK293T cells. α-Tubulin was used as a loading control. **D.**-**F.** Mutation of Ser885 to alanine (S885A) in Par3 prevents SIK-mediated degradation (D), while mutation of Ser888 (E) or Ser884 (F) to alanine has no effect. WT SIK was co-expressed with either WT or mutated full-length Par3 in HEK293T cells. α-Tubulin was used as a loading control (D, F), and protein levels in (E) were normalized with a Bradford assay. Densitometric quantification is provided on top of the Par3 and SIK immunoblot lanes in each panel; the values indicate relative expression of each protein normalized to the corresponding level of α-tubulin (panels B, C, D and F) or absolute densitometric values after subtraction of background density (panel E). The basal levels corresponding to endogenous expression of each protein show small variability from one panel to the other, which reflects protein expression and stability in each independent experiment.

We started validation of the bioinformatic result by first asking whether SIK could associate with its putative substrate, Par3. HEK239T cells were transfected with the three isoforms of mouse Par3 (here called Par3 100 kD, 150 kD and 180 kD, see also Figure 2A), in the presence or absence of co-expressed SIK (Figure 1A). Upon immunoprecipitation of Par3, we observed significant co-precipitation of endogenous SIK with the 150 kD Par3 isoform and less with the 100 and 180 kD isoforms (Figure 1A, minus SIK panel); transfected SIK co-precipitated more efficiently with the three Par3 isoforms (Figure 1A). Control immunoprecipitations with a non-immune rabbit immunoglobulin resulted in weak background signals (Figure 1A). We also performed the inverse immunoprecipitation using a home-made SIK antibody and HEK293T cells transfected with the catalytically inactive SIK K56R mutant (Figure 1B). Transfection of the kinase-dead variant of SIK was chosen to prevent the rapid degradation of the kinase-active SIK itself as previously established [34]. Upon immunoprecipitation of SIK K56R, a stable complex with all Par3 isoforms could be detected; the interaction with the 100 kD isoform of Par3 appeared stronger, which may reflect the fact that the 100 kD Par3 isoform was expressed at a much higher level compared to the 150 kD and 180 kD isoforms (Figure 1A, B).

We then wanted to provide evidence for the endogenous complex between SIK and Par3. However, as we established previously [34], the endogenous levels of SIK are very low as this protein constantly turns over, creating a practical difficulty in measuring transient protein associations. To circumvent this limitation, we expressed exogenous SIK and monitored the formation of complexes with endogenous Par3 in mouse mammary epithelial NMuMG cells. Both wild-type and kinase-inactive (K56R) forms of SIK were transfected in these experiments; upon immunoprecipitation of SIK, we observed a protein complex between endogenous (180 kD isoform) Par3 and wild-type SIK, and an even more enhanced complex with the catalytically inactive SIK (Figure 1C). The inverse co-immunoprecipitation confirmed this result since only a very weak complex was seen between Par3 and wild-type SIK, while a much stronger complex was detected between endogenous Par3 and inactive SIK (Figure 1C). In the same experiment, we also exposed the cells to long-term stimulation with TGFβ, a cytokine that causes robust EMT in NMuMG cells (see below). Upon TGFβ stimulation, the SIK-Par3 complexes were again detected, albeit at lower levels.

We next addressed whether SIK binding to Par3 affects the phosphorylation status of Par3. To this end, we immunoprecipitated endogenous Par3 from NMuMG mammary epithelial cells and performed *in vitro* phosphorylation assays using radioactive [^32^P]ATP (Figure 1D). The Par3 immunoprecipitates demonstrated significant levels of incorporation of the radioactive phosphate in proteins that corresponded to the size of Par3 (180 kD isoform, top band in Figure 1D), whereas precipitation with control immunoglobulin gave only background radioactive signals without specific protein bands (Figure 1D). When the cells were transfected with siRNA that silences endogenous SIK (siSIK), the amount of incorporated radioactivity in the Par3 protein band was significantly decreased (Figure 1D), suggesting that endogenous SIK co-precipitated with Par3 and facilitated phosphorylation of Par3. In summary, these results establish the ability of SIK to associate with Par3 and further support the notion that SIK can promote phosphorylation of Par3.

### SIK drives degradation of Par3 in a Ser885-dependent manner

We next investigated the functional role of the SIK-Par3 interaction. During the co-immunoprecipitation experiments, we observed that when wild-type SIK was co-expressed with Par3, the levels of the 150 and 180 kD isoforms decreased (Figure 1A). Moreover, when HEK239T cells were transfected with full-length Par3 in the presence of increasing amount of SIK, Par3 was downregulated to roughly 50% of its starting level (Figure 2B). The downregulation of Par3 levels by SIK depended on the catalytic activity of the kinase, as the K56R mutant SIK failed completely or in certain experiments could only weakly downregulate Par3 (Figure 2C). Mutating Ser885 in mouse Par3 to an alanine residue rendered Par3 significantly resistant to the negative effect exhibited by SIK (Figure 2D-2F), suggesting that the predicted phosphorylation site in Par3 is functionally relevant. In contrast, mutagenesis of the neighboring Ser884 or Ser888 to alanine residues did not rescue Par3 downregulation by SIK (Figure 2E, 2F), demonstrating high sequence specificity within the short serine-rich motif of Par3 (Figure 2A).

**Figure 3:**
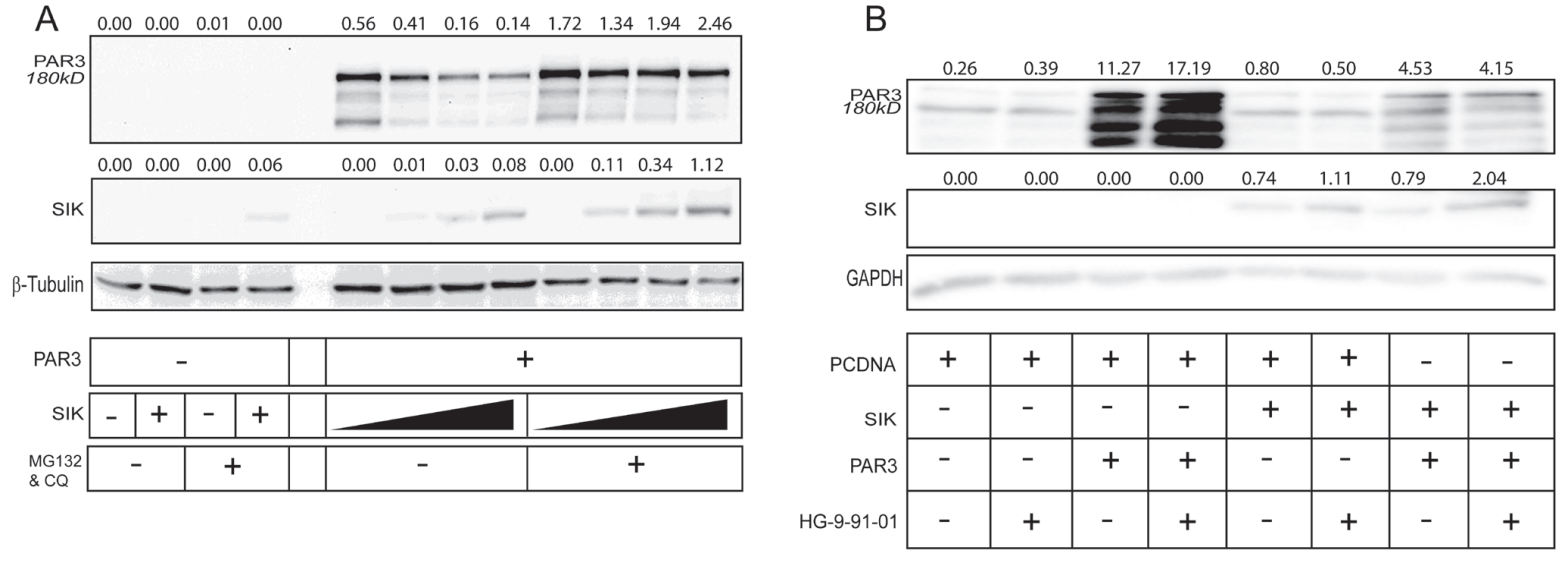
Proteasomal, lysosomal and SIK inhibitors stabilize Par3 **A.** HEK293T cells were co-transfected with increasing amount of WT SIK and constant amount of Par3 in the presence and absence of MG132 and chloroquine (CQ) to inhibit proteasomal and lysosomal protease activity respectively, followed by immunoblotting of total cell lysates. α-Tubulin was used as a loading control. **B.** HEK293T cells were co-transfected with WT SIK and Par3 in the presence and absence of the pan-SIK inhibitor HG-9-91-01 for a total period of 36 h, followed by immunoblotting of total cell lysates. GAPDH was used as a loading control. Representative immunoblots out of at least three repeats are shown in each panel. Densitometric quantification is provided on top of the Par3 and SIK immunoblot lanes in each panel; the values indicate relative expression of each protein normalized to the corresponding level of α-tubulin (panel A) or GAPDH (panel B). The basal levels corresponding to endogenous expression of each protein show small variability from one panel to the other, which reflects protein expression and stability in each independent experiment. Molecular size markers in kilo Dalton (kD) are also marked.

In order to investigate whether Par3 was degraded via proteasomes, we repeated the dose-dependent SIK transfection experiments in the presence of the proteasomal inhibitor MG132; we observed a weak, but reproducible stabilization of Par3 (data not shown). However, when we treated cells with both MG132 and the lysosomal inhibitor chloroquine, Par3 was stabilized at a high level despite the high expression of wild-type SIK (Figure 3A). It is worth noting that MG132 and chloroquine also stabilized SIK (Figure 3A). Attempts to also demonstrate enhanced ubiquitination of Par3 after SIK co-expression did not succeed, as we encountered technical difficulty in monitoring the ubiquitination of high molecular size Par3 180 kD (data not shown). We then employed the established chemical inhibitor of SIK family kinase activity HG-9-91-01 [46]. In HEK293T cells, the transfected 180 kD Par3 protein band was stabilized and enhanced in the presence of HG-9-91-01 (Figure 3B). The reduction of Par3 levels induced by co-expression of SIK was reverted by the HG-9-91-01 inhibitor, and simultaneously the transfected SIK level was enhanced (Figure 3B). Thus, although this inhibitor enhanced SIK protein stability, it partially blocked the reduction in Par3 180 kD levels. These results propose that SIK promotes Par3 phosphorylation on Ser885, which leads to downregulation of Par3 via degradation in proteasomes and/or lysosomes.

### SIK regulates tight junction degradation

**Figure 4:**
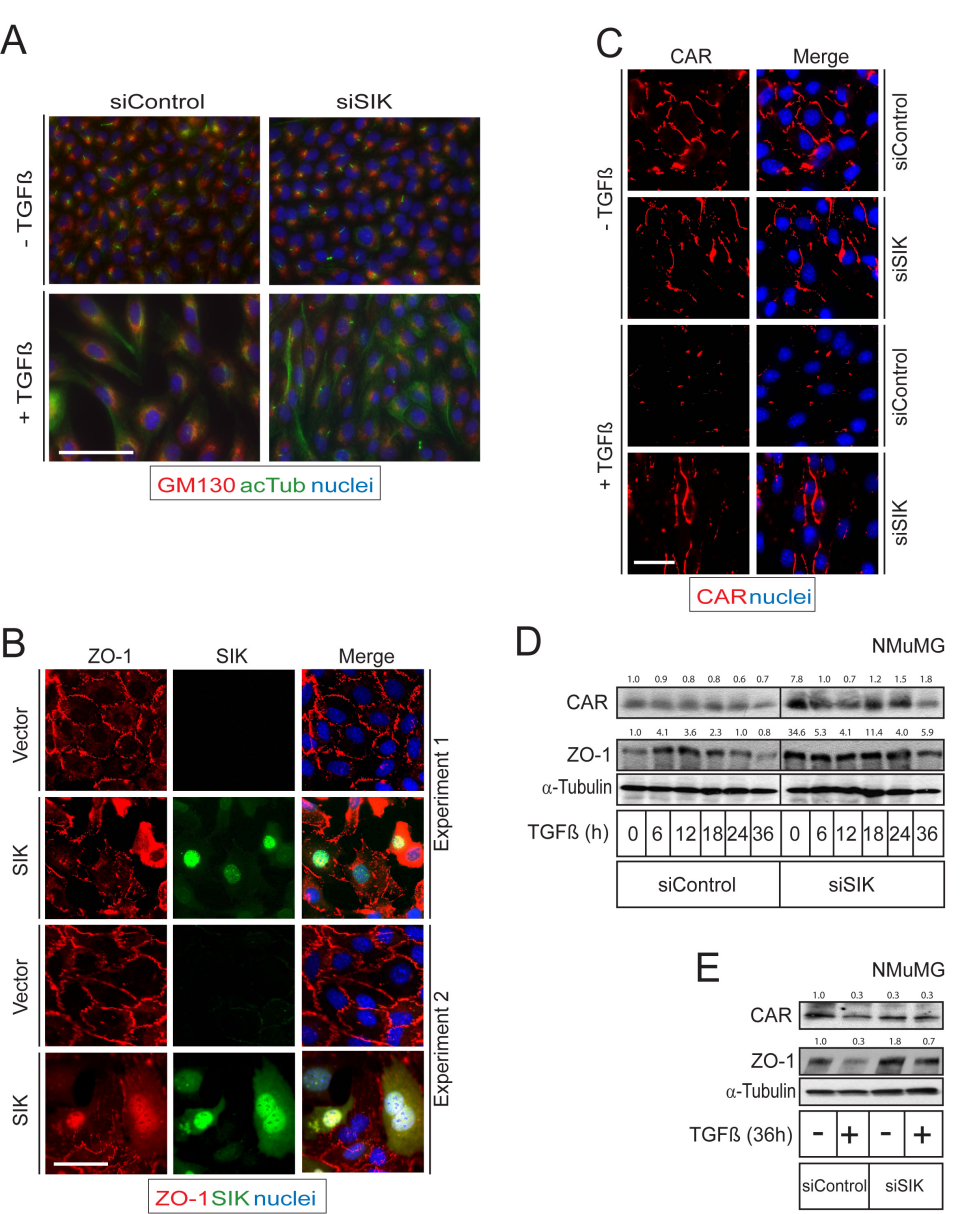
SIK affects polarization and TJ stability **A.** NMuMG cells were transfected with siControl and siSIK followed by TGFβ stimulation. Two polarity markers GM130 (marker for the Golgi apparatus, in red) and acetylated tubulin (marker for the cilium, in green) were used. **B.** Immunofluorescence analysis after viral transduction of Flag-tagged SIK in NMuMG cells. Cells were infected with Flag-tagged SIK adenovirus for 6 h, after which the virus was removed from the cells. Two independent microscopic fields representative for the experiment are shown after immunostaining for ZO-1 and SIK; the merged pictures also include nuclear DAPI staining. **C.** Immunofluorescence microscopy showing TJ stabilization during TGFβ-induced EMT. The cells were transfected with siControl or siSIK twice, with a 24 h interval between transfections, and were stimulated with TGFβ (5 ng/ml) 36 h after the second transfection. A representative experiment is shown after immunostaining for CAR; the merged pictures also include nuclear DAPI staining. Bars indicate 10 µm in panels A-C. **D.** Immunoblot showing the levels of two TJ proteins (CAR and ZO-1) showing stabilization of the TJ during TGFβ stimulation in NMuMG cells. Knockdown of SIK was performed as described in (C) and cells were treated with TGFβ (5 ng/ml) or vehicle for the indicated times. α-Tubulin was used as a loading control. **E.** Immunoblot showing the protein levels corresponding to the experiment in (C). Densitometric quantification is provided on top of the CAR and ZO-1 immunoblot lanes in each panel (D, E); the values indicate relative expression of each protein normalized to the corresponding level of α-tubulin, and further normalization relative to the control condition of siControl in the absence of stimulation with TGFβ (first lane in all immunoblots, expressed with the basal value of 1.0). Representative immunofluorescence microscopy images and immunoblots out of at least three repeats are shown in each panel.

Since Par3 is a major regulator of epithelial polarity, we postulated that downregulation of Par3 by the action of SIK could be linked to changes in epithelial differentiation. A cellular process that involves changes in cell polarity is EMT, which is promoted by many growth factors, including TGFβ [11, 47]. We chose to work with the NMuMG mouse mammary epithelial cells because they represent a well-established model where TGFβ potently downregulates AJ and TJ and activates the TGFβ receptor-Par6 signaling pathway [15, 48]. We stained cells with markers of cell polarity, such as the Golgi apparatus protein GP130 that showed Golgi localization only on one side of the nucleus, and acetylated tubulin that decorates the cilium, which showed localization at the opposite pole of the nucleus relative to the Golgi, i.e. at the centrosome (Figure 4A) [49]. Upon stimulation with TGFβ, the NMuMG cells transit towards a mesenchymal phenotype and re-organize their polarity; accordingly, the Golgi apparatus formed a complete ring around the nucleus, whereas the cilium disappeared and acetylated tubulin decorated an extended microtubular network within the cytoplasm after TGFβ stimulation (Figure 4A). Interestingly, silencing of endogenous SIK resulted in an overall more polarized pattern whereby the Golgi staining was confined to a smaller spot on one side of the nucleus and the cilium with acetylated tubulin was also easier to observe on every cell; the result of SIK silencing was more pronounced after TGFβ stimulation since the cells restricted the distribution of their Golgi to a smaller area on a single side of the nucleus, and most cells formed distinct cilia despite the presence of diffuse acetylated microtubular networks in a distinctly visible number of the cells (Figure 4A). These results suggest that endogenous SIK contributes to the TGFβ-mediated de-polarization of epithelial cells such as NMuMG.

During TGFβ signaling the TGFβRII phosphorylates Par6, another polarity protein, leading to recruitment of the ubiquitin ligase Smurf1 and degradation of the small GTPase RhoA [15]. The net result of this mechanism is degradation of TJs. Our findings suggested that SIK may have a role in TJ degradation. ZO-1 is a member of the zona occludens family of adaptor proteins that assemble the TJs, which is downregulated after stimulation with TGFβ [15]. In order to further explore whether SIK controls TJs, we examined ZO-1 downregulation under control conditions or after expressing high levels of SIK by means of adenoviral infection. ZO-1-positive TJs were dramatically disintegrated in cells expressing SIK, while neighboring cells that expressed undetectable levels of SIK or cells infected with control virus exhibited well assembled TJs (Figure 4B). SIK localizes to both the nucleus and the cytoplasm, and its shuttling can be regulated by the 14-3-3 signaling protein during cAMP and calcium signaling [27, 50]. When overexpressed, both a nuclear localization and a diffuse cytoplasmic distribution could be observed, including localization at the plasma membrane (Figure 4B) [23, 34]. We performed similar experiments by analyzing the activity of endogenous SIK and measuring TJ (and AJs) using the SIK chemical inhibitor HG-9-91-01 (Supplementary Figure 2). Treating NMuMG cells with HG-9-91-01 for 24 h resulted in a relative stabilization of the endogenous levels of ZO-1, E-cadherin, Par3 and SIK (Supplementary Figure 2). Accordingly, this inhibitor not only affected Par3 stability and the associated TJ protein ZO-1, but even affected the major AJ protein E-cadherin. The stabilizing effect was evident in spite of the concomitant stabilization of the endogenous SIK protein (Supplementary Figure 2).

In order to understand the role of SIK in regulating TJs under more physiological conditions, we induced TJ disassembly by stimulating the cells with TGFβ while performing siRNA-mediated silencing of endogenous SIK (Figure 4C-4E). The levels of both basal and TGFβ-induced SIK protein were effectively reduced by 70-80% after transfection of siRNA, as determined by *SIK* mRNA analysis (Supplementary Figure 3A). TJ complexes were assessed by immunofluorescence staining for ZO-1 (not shown) and CAR, another structural member of the TJ that passes the plasma membrane and whose levels are reduced upon TGFβ stimulation [51]. Cells transfected with control siRNA completely lost assembled TJs from their cell surface when stimulated with TGFβ; in contrast, after silencing of endogenous SIK, many of the TJs remained intact and were not downregulated by TGFβ stimulation (Figure 4C-4E).

SIK is known to function as a repressor of CREB-mediated transcription [36] and we therefore considered the possibility that SIK might regulate TJs at the transcriptional level. This possibility was also supported by the nuclear localization of SIK (Figure 4B). NMuMG cells, treated with siSIK or control siRNA, were stimulated with TGFβ for different time periods. Next we measured the levels of CAR and ZO-1 mRNA (Supplementary Figure 3B, C), the protein levels of which were affected by SIK (Figure 4B, 4C). No significant effect of SIK downregulation on the mRNA of CAR and ZO-1 was observed (Supplementary Figure 3B, C). From these observations we conclude that SIK mediates downregulation of TJs, primarily by destabilizing TJ proteins, and not by affecting their transcription.

To provide more quantitative measurement of the role of endogenous SIK in TJ protein downregulation during TGFβ signaling, we repeated the above experiment and analyzed cell extracts for expression of the TJ proteins ZO-1 and CAR (Figure 4D, 4E). Time course experiments showed that TGFβ induced downregulation of CAR (down to 40% of basal levels) and ZO-1 (down to 20% of basal levels) protein levels under control conditions, while silencing of SIK by siRNA led to significant 8- and 30-fold stabilization of the CAR and ZO-1 levels respectively (Figure 4D). TGFβ stimulation in the presence of SIK silencing led to time-dependent CAR and ZO-1 downregulation, however, CAR and ZO-1 protein levels remained higher than the corresponding levels in the presence of control silencing throughout the time course experiment (Figure 4D), and even after prolonged (36 h) stimulation with TGFβ (Figure 4E). We therefore conclude that SIK promotes the dissolution of TJs during TGFβ signaling.

### Downregulation of Par3 in response to TGFβ

**Figure 5:**
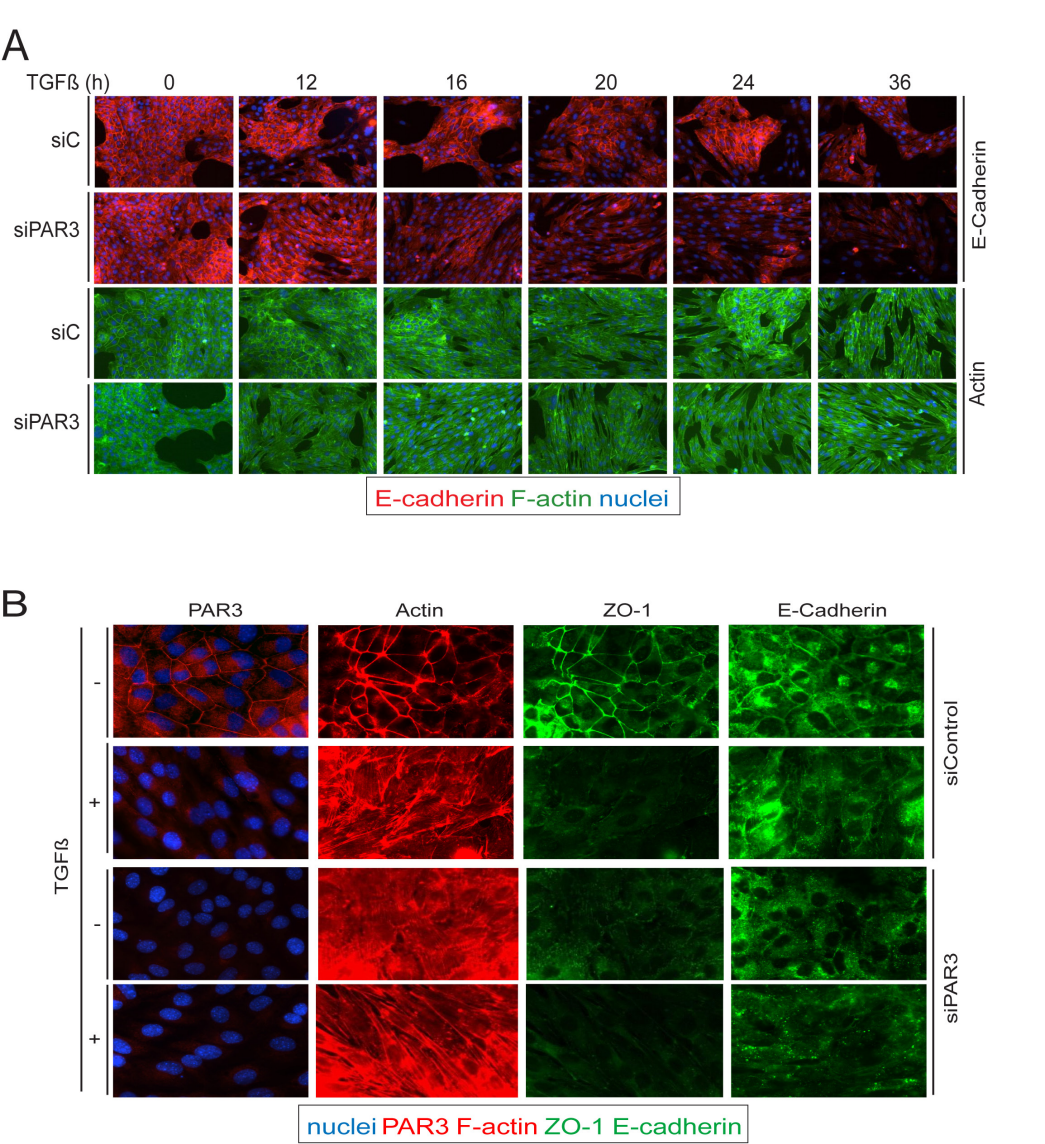
Par3 downregulation precipitates loss of cell-cell junctions in response to TGFβ **A.**, **B.** Comparison of cell junctions and actin cytoskeleton between siControl and siSIK transfected cells. A. NMuMG cells were transfected with the corresponding siRNAs 24 h prior and at the start of TGFβ stimulation (5 ng/ml). Changes of E-cadherin (E-cadh) expression and localization (red) as well as changes in the actin cytoskeleton (green) were observed for the indicated times of stimulation. B. NMuMG cells were transfected with siControl or siPar3 as in (A), and cells were incubated with TGFβ (5 ng/ml) or vehicle for 36 h. Par3, ZO-1 and E-cadherin expression and localization are shown, as well as changes in the actin cytoskeleton.

Since TGFβ signaling leads to induction of SIK levels [23, 34], and since we found that SIK leads to downregulation of Par3, we wanted to explore the possibility that loss of Par3 could destabilize AJ and TJ in NMuMG cells. Time course experiments of TGFβ stimulation followed by immunofluorescence analysis for E-cadherin and direct fluorescence microscopy for actin showed that under control conditions, TGFβ induced loss of AJs and actin reorganization from a cortical assembly to stress fibers after 36 h of stimulation (Figure 5A). Interestingly, after silencing of endogenous Par3, the response to TGFβ was dramatically accelerated and was complete only 16 h after TGFβ stimulation. Immunostaining for endogenous Par3 confirmed that TGFβ downregulated endogenous Par3 to the same extent as ZO-1 during the 36 h interval of TGFβ signaling (Figure 5B). In this experiment we also confirmed that silencing of Par3 exhibited complete loss of the endogenous protein with concomitant complete disassembly of ZO-1-positive TJs, even in the absence of TGFβ stimulation (Figure 5B). We therefore conclude that Par3 downregulation leads to a more pronounced loss of AJ and TJ, which correlates well with the proposed role of SIK in mammary epithelial junctional disassembly.

### High SIK and low Par3 expression in human cancer

**Figure 6:**
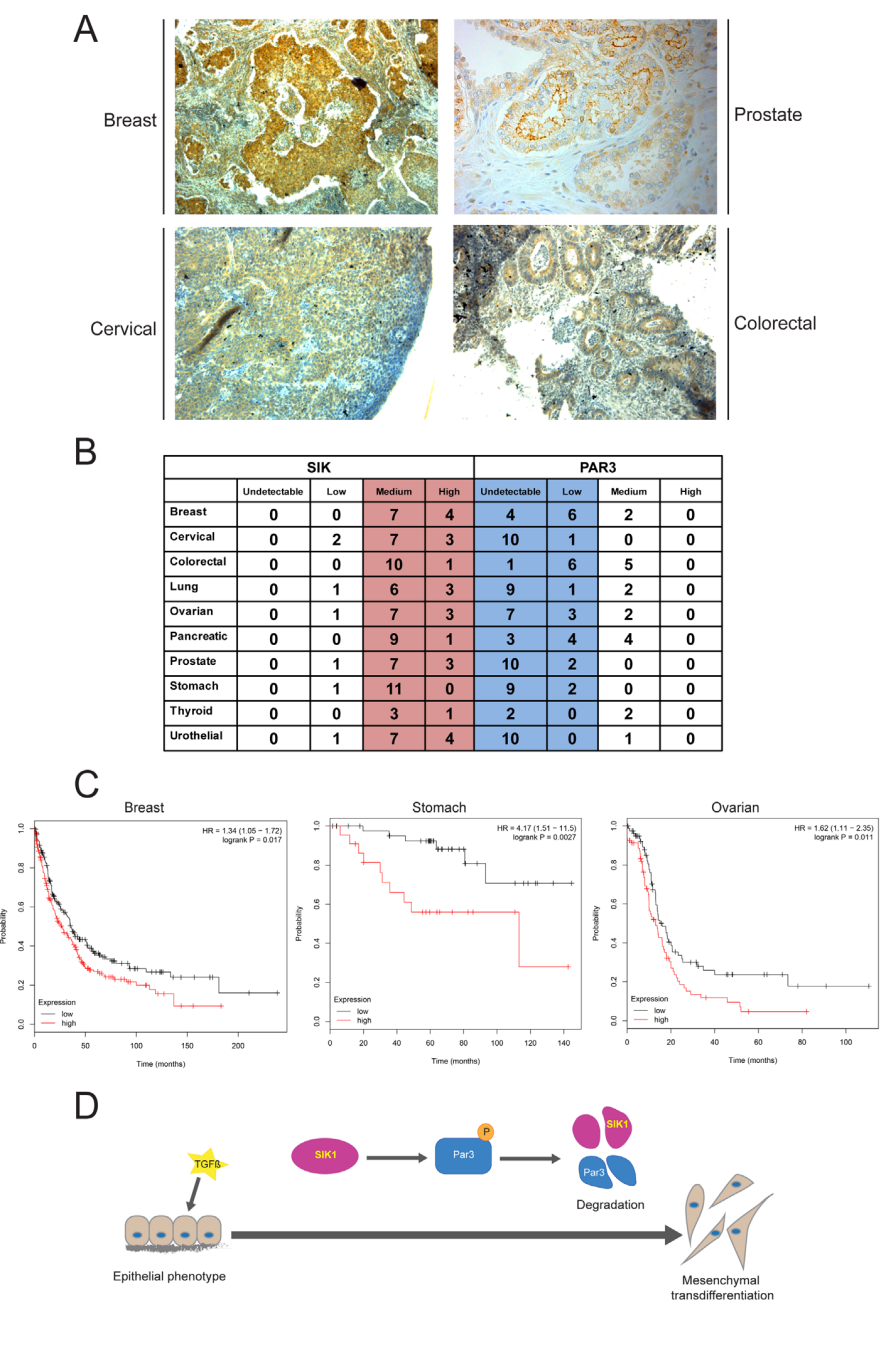
Expression of SIK and Par3 negatively correlate in human tumors **A.** Immunohistochemical analysis of expression of SIK (brown staining) in ductal breast carcinoma, Gleason grade 3 prostate carcinoma, cervical carcinoma and colorectal carcinoma. Tumor tissue was counterstained with hematoxylin. **B.** Table of SIK and Par3 protein expression in various human tumors analyzed by immunohistochemistry. The data are derived from the human protein atlas (https://www.proteinatlas.org/) and the number of individual patient samples per tumor type (left column) is plotted under four different categories, undetectable, low, medium and high. Note that in all 10 tumor types relatively high (medium and high) SIK expression corresponds to significantly low (low and medium) Par3 expression. The average number of patient specimen analyzed were between 10 and 12 for most tumors except for thyroid carcinoma where 4 independent patient samples were scored. **C.** Kaplan-Meier survival curves of breast, stomach (gastric) and ovarian carcinoma patients with low (black curves) and high (red curves) levels of *SIK* mRNA expression according to TCGA dataset. Statistical significance of the difference between the two curves is also indicated in terms of the corresponding hazard ratio (HR) with 95% confidence interval and associated probability value, which is smaller than 0.02, based on a χ-squared statistic. **D.** Overview of SIK-mediated effects on Par3. TGFβ acts on epithelial cells causing SIK expression, which then promotes Par3 phosphorylation (P symbol in circle), causing degradation of both Par3 and SIK1. During the same course of TGFβ signaling epithelial cells transdifferentiate (thick arrow) to mesenchymal.

In order to investigate the possible relevance of our findings to human cancer, we examined expression of SIK in human cancer specimen (Figure 6A). SIK was ubiquitously expressed in the cytoplasm (and less in the nucleus) of cancer cells in breast, prostate, cervical and colorectal carcinomas. In all these tumor tissues, the cytoplasmic distribution of SIK showed a clear septate pattern corresponding to cell-cell junctions (Figure 6A). This distribution is compatible with a role of SIK as a regulator of TJ assembly. In order to correlate SIK protein expression to Par3 levels in a broader spectrum of human tumors, we analyzed the human protein atlas database (HPA, https://www.proteinatlas.org/), in which our home-made anti-SIK antibody [34] was widely used for immunohistochemical analyses of normal and cancer tissues. In 10 major groups of carcinomas, examination of tumor tissue in 10-12 patient specimen reproducibly revealed relatively high SIK expression, whereas the same specimen exhibited low or undetectable levels of Par3 (Figure 6B, red *versus* blue shading). Although this analysis was not based on serial sections of the exact same tumor piece stained for the two different proteins, the overall and unbiased (each protein staining was performed independent from the other) immunohistochemical pattern fits to the model proposed here. We then chose human breast carcinoma to explore the correlation of the expression levels of SIK with the levels of Par3 and the activity of the TGFβ pathway, measured as levels of phosphorylated Smad2 in the tumor tissue (Supplementary Figure 4A). Immunostaining of serial sections from patients showed that cytoplasmic SIK expression in the carcinoma compartment correlated with the expression of phosphorylated Smad2 levels and with complete absence of Par3. Interestingly, the surrounding stromal tissue was completely negative for SIK and phosphorylated Smad2 and was positive for Par3 (Supplementary Figure 4A). In the same breast carcinoma sections we also performed DNA ploidy analysis with the aim of correlating SIK expression to the state of genomic stability of the tumor. Low nuclear signal of SIK was observed in breast cancer cases with unstable aneuploid, diploid and tetraploid DNA content (*p* = 0.022) compared to the cases with diploid stable prognostically favorable DNA measure (low nuclear signal in Supplementary Figure 4B).

Metastatic dissemination is often the terminal stage of cancer progression. SIK has previously been demonstrated to act as a metastasis suppressor based on a cellular mechanism whereby SIK induced p53-dependent anoikis [33]. We queried a variety of human carcinomas for evidence of the prognostic value of impaired expression of SIK on overall survival using the Kaplan-Meier plotter web-tool (see Method). We identified human carcinomas where high level of *SIK* mRNA expression correlated with worse disease outcome, in other words, lower chance of survival after progressed disease stage, as exemplified by breast, gastric and ovarian carcinomas (Figure 6C). This unbiased analysis in an even larger cohort of cancer patients (provided by the combined data sets of the cancer genome atlas (TCGA), gene expression omnibus (GEO) and the European genome-phenome archive (EGA), revealed another indication that high SIK expression best correlates with tumor aggressiveness and unfavorable disease outcome (Figure 6C). Thus, analysis of human cancer at multiple levels supports the cellular and molecular model derived from studies in NMuMG cells and suggests that SIK expression can be regulated in epithelial cancers and may also have significant functional repercussions.

## DISCUSSION

Recently, we established a role for SIK in a negative feedback loop during TGFβ signaling [23, 34]. Here we have explored new possible functions of this kinase. We found that SIK plays a role in mediating tight junction stability by degrading Par3, a key member of the polarity complex (Figure 2). We observed that knockdown of endogenous SIK resulted in enhanced stability of the TJ complex and partial resistance against TGFβ-induced depolarization of the Golgi apparatus and dissolution of the epithelial cilium, while overexpression of SIK led to enhanced degradation of the TJs (Figure 4). We also demonstrated that SIK interacts with Par3 (Figure 1) and that Ser885 in the Par3 aPKC-binding domain, when mutated to alanine, confers resistance to the effects of SIK (Figure 2). Finally, the correlation between high SIK levels and low Par3 levels established by experiments using cultured epithelial cells (Figures 2, 4), also reflects the expression patterns of the same proteins in advanced human carcinomas (Figure 6, Supplementary Figure 4). The combined evidence from the current study and our previous work establishes a model whereby TGFβ signaling rapidly induces expression of the unstable kinase SIK, leading to its transient accumulation. SIK then associates with and targets Par3 and thus mediates the downregulation of TJ (Figure 6D). In parallel, SIK can associate with Smad7 to downregulate the TGFβ receptor, leading to negative feedback regulation [23, 34]. The new evidence (Figure 6D), when combined with our previous findings [23, 34], proposes that the process of epithelial junctional assembly is molecularly linked to the mechanism of TGFβ signaling downregulation. Interestingly, studies of kidney fibrosis in a model of diabetic nephropathy where TGFβ-dependent accumulation of extracellular matrix is a key feature of the fibrotic phenotype, corroborated the above model [32]. In mesangial cells, high glucose caused SIK downregulation, subsequent TGFβRI upregulation and activation leading to fibrosis, whereas, SIK activation resulted in TGFβRI degradation and normalization of the fibrotic phenotype [32].

The model we propose linking SIK function to TGFβ signaling, TJ and polarity complex protein stability, suggests that SIK may act as a pro-tumorigenic or pro-invasive factor during cancer progression. However, TGFβ signaling is well known for its context-dependent actions [4]; during cancer progression, TGFβ acts as both a tumor suppressor and a pro-tumorigenic, pro-metastatic factor [52]. Thus, it is possible that TGFβ-induced SIK expression is involved in cytostatic, pro-apoptotic or tumor suppressor mechanisms during specific stages of cancer evolution. In agreement with this notion, silencing of SIK in immortalized mammary epithelial cells demonstrated that SIK suppresses the anchorage-independent growth of these cells [33]. Furthermore, the LKB1-SIK signaling module was linked to p53-mediated cell death under non-adherent conditions, i.e. anoikis [33]. In the same study, silencing of SIK dramatically suppressed primary tumorigenesis in an orthotopic breast cancer model, suggesting a pro-oncogenic action of SIK, however, the rare primary tumors that developed under low SIK expression conditions, exhibited a dramatic increase in metastatic incidence, suggesting a role for SIK as a metastasis suppressor [33]. This evidence is further enhanced by studies on the EMT in lung and liver cancer and the analysis of various transcriptional mechanisms mediated by SIK [53-55]. In non-small cell lung carcinoma cells with overt signs of EMT and resistance to radiation therapy, weak signaling by LKB1 and SIK kinases could be measured, resulting in derepression of the EMT transcription factor ZEB1 [55]. Whereas loss of LKB1 convincingly correlates with EMT and cancer cell invasiveness, a role for SIK remains to be established [55]. SIK expression is downregulated in aggressive forms of hepatocellular carcinoma (HCC) [53]. HCC with SIK overexpression also had high levels of E-cadherin and ZO-1 proteins, whereas silencing of SIK downregulated these epithelial junctional proteins [53]. In the HCC model, SIK would phosphorylate a transcriptional co-repressor protein of β-catenin, inhibiting the β-catenin/TCF4 complex from inducing the EMT transcription factor Twist1 [53]. For this reason, silencing of SIK would promote a transcriptionally active β-catenin/TCF4 complex, leading to high Twist1 levels and EMT [53]. Twist1 would also transcriptionally repress the *SIK* gene, explaining the low SIK expression in aggressive HCCs [53]. Furthermore, the low levels of SIK protein in aggressive HCC are maintained by the nuclear ubiquitin ligase RNF2, a subunit of the Polycomb co-repressor complex 1 [54]. Additional studies in lung and kidney epithelial cells support the nuclear actions of SIK. Silencing of SIK activated CREB, leading to misexpression of Snail2, ZEB1/2 and Twist1, and repression of E-cadherin [56].

The above nuclear mechanisms appear to differ from the model generated in this paper based on regulation of Par3 by SIK in the cytoplasm (Figure 6D). We failed to observe robust effects of SIK on epithelial gene mRNA expression in response to TGFβ stimulation (Supplementary Figure 3B, C). On the other hand, the chemical inhibitor HG-9-91-01 stabilized E-cadherin protein levels in NMuMG cells in the absence of TGFβ stimulation (Supplementary Figure 2). Considering the possibility that more predicted substrates of SIK phosphorylation (Table 1) are verified experimentally, proteins such as SIPA1, CTNND2, SSH1, CGN, ABLIM1, RACGAP1 and PSD3, provide additional regulatory mechanisms by which SIK may impact on the assembly of epithelial junctions, including mechanisms of dynamic reorganization of the cytoskeleton, which is frequently observed during junctional remodeling. Our observations emphasize a cytoplasmic role of SIK, based on protein complexes with Par3 (Figure 1) and immunolocalization at cell-cell junctions in tumor specimen (Figure 6). The analysis of human cancer specimen (Figure 6, Supplementary Figure 4) corroborates the molecular model presented here. The SIK antibody that we generated has been deposited to the Human Protein Atlas database, and helped confirm widespread expression of SIK in human tumors of diverse tissue origins (www.proteinatlas.org/ENSG00000142178-SIK1). Analysis of such data revealed high SIK expression in several carcinomas (Figure 6B). Furthermore, survival analysis in cancer patients provided a negative correlation of high *SIK* mRNA expression and chance for patient overall survival (Figure 6C). In addition, nuclear SIK localization in breast cancer was related to such a criterion of favorable prognosis as genomic stability (Supplementary Figure 4B), supporting a hypothesis whereby cytoplasmic SIK may contribute to detrimental outcome of cancer progression. The collective evidence provided by this study argues strongly for a pro-tumorigenic role of SIK in various carcinomas. We therefore propose that similar to TGFβ signaling, SIK may have bi-phasic and distinct functions, in the cytoplasm and the nucleus. It will therefore be important to analyze further the relative contributions of SIK during cancer progression and define those instances where SIK operates under the control of TGFβ signaling or under the control of alternative signaling pathways, thus possibly promoting tumor progression or even suppressing tumorigenesis.

Although the evidence that we present here only suggests that SIK promotes Par3 phosphorylation, the location of Ser885 in a functionally important domain of Par3 (Figure 2A), implies that SIK may cause the dissociation of aPKC from Par3. In addition, the physiological relevance of phosphorylation of Par3 on Ser885 requires further analysis using phospho-specific antibodies for this site, and a thorough screen against multiple protein kinases that could mediate a direct chemical modification. Our observations so far indicate that Par3 is degraded by both proteasomes and lysosomes (Figure 3A), and this is compatible with a model where multiple components of the polarity complex are proteolytically degraded together with associated tight junctional complexes.

Loss of Par3 has been connected to defective recruitment of the polarity complex to junctions, leading to failure in the maturation step of junctional complex assembly [57, 58]. It has been shown that Par3 downregulation is mediated by miRNA 491 and is a necessary step for TGFβ-induced EMT [59, 60]. In this paper, we propose a mechanism by which TGFβ could achieve degradation of Par3 through induction of SIK, which complements the miRNA-mediated mechanism. In *Drosophila,* phosphorylation of Bazooka, the homolog of Par3, by Par1 is known to disrupt formation of the Par3/Par6/aPKC complex, leading to depolarization of cells [61, 62]. This scenario resembles the model proposed here where SIK affects the stability of Par3. Interestingly, another member of the polarity complex, Par6, has been shown to interact with TGFβRI, become phosphorylated by the TGFβRII, and cause degradation of RhoA by Smurf1-mediated ubiquitination [15]. Since we have shown that SIK also binds to TGFβRI and together with Smurf2 promotes degradation of TGFβRI [23], we consider favorably the possibility of involvement of Smurf E3 ligases in the degradation mechanism of Par3, opening up an exciting scenario of coordinate TJ and polarity regulation by ubiquitination. On the other hand, RNF2 that ubiquitinates and degrades SIK [54], may also be involved in Par3 degradation, assuming that an unknown mechanism enforces cytoplasmic residence of RNF2; the latter is compatible with our observations that support coordinate degradation of SIK and Par3 (Figure 3, 4).

In conclusion, SIK provides different ways of regulation during TGFβ signaling, by influencing TJ and polarity complex members. Further exploration into the details by which SIK leads to degradation of its targets is warranted. The involvement of enzymes, like SIK, also offers the future prospect for exploitation of pharmaceutical inhibition of specific subprograms of TGFβ signaling that may be of interest in the treatment of cancer or fibrotic diseases.

## MATERIALS AND METHODS

### Cells, plasmids and siRNAs

Human HaCaT keratinocytes, human embryonic kidney HEK293 and HEK293T cells, murine mammary epithelial NMuMG cells and green monkey kidney COS1 cells, and culture conditions, were as described [22, 34].

Expression vectors pcDNA3-Flag-hSIK and the SIK ATP-binding site mutant K56R were described before [34]. The vectors pcDNA-Par3 100, 150 and 180 kD were a kind gift from P. Aspenström, Karolinska Institute, Stockholm, and described by Lin et al [63]. All DNA constructs were sequence verified.

Mouse *SIK*-specific siRNA, ON-TARGETplus SMARTpool reagent L-0044399-00 (Acc. No. NM_010831), and control siRNA against the *luciferase* reporter vector pGL2 (Acc. No. X65324) were from Dharmacon Research, Inc., Boulder, CO.

### Growth factors and chemical reagents

Recombinant mature human TGFβ1 was bought from PeproTech EC Ltd. (London, UK) and Biosource Inc. (Camarillo, CA, USA). The TGFβ1 isoform was used throughout this study and is referred to as TGFβ, was dissolved in vehicle consisting 4 mM HCl/0.1% (w/v) fatty acid-free bovine serum albumin. The pan-SIK chemical inhibitor HG-9-91-01 (Cayman Chemical, Ann Arbor) was dissolved in dimethylsulfoxide and was used at concentrations between 0.5 and 100 µM. The proteasomal inhibitor MG132 (Sigma-Aldrich Sweden AB), was dissolved in dimethylsulfoxide and was used at a concentration of 50 µM, and the lysosomal inhibitor chloroquine (Calbiochem/Merck Chemicals and Life Science AB, Stockholm), dissolved in water, was used at 40 µg/ml.

### Antibodies

Mouse anti-Flag (M5) antibody (catalogue number F4042), purchased from Sigma-Aldrich Sweden AB (Täby, Sweden) and used at 1:1,000 v/v dilution; the specificity of the anti-Flag antibody was verified based on the recognition of the appropriate transfected protein and gave rise to some background bands that were easy to discriminate against. Mouse anti-acetylated α-tubulin antibody (clone 6-11B-1, catalogue number T7451), purchased from Sigma-Aldrich Sweden AB (Täby, Sweden) and used at 1:500 v/v dilution; the specificity of the antibody was verified based on the molecular size of the single protein band identified by immunoblotting and the co-staining of microtubules in immunofluorescence experiments. Mouse monoclonal anti-α-tubulin antibody (catalogue number sc-8035), purchased from Santa Cruz Inc. (Santa Cruz, CA, USA) and used at 1:1,000 v/v dilution; the specificity of the antibody was verified based on the electrophoretic mobility of the protein appearing as single band and the co-staining of microtubules in immunofluorescence experiments. Mouse monoclonal anti-GAPDH antibody (catalogue number AM4300), purchased from Ambion, Thermofisher Scientific (Stockholm, Sweden) and used at 1:50,000 v/v dilution; the specificity of the antibody was verified based on the electrophoretic mobility of the protein appearing as single band. Rabbit anti-GM130 polyclonal antibody (affinity-purified, catalogue number G7295), purchased from Sigma-Aldrich Sweden AB (Täby, Sweden) and used at 1:1,000 v/v dilution; the specificity of the antibody was verified based on the molecular size of the single protein band identified by immunoblotting and the staining of perinuclear Golgi apparatus in immunofluorescence experiments. Mouse anti-ZO-1 monoclonal antibody (catalogue number 33-9100) was obtained from Invitrogen/Life Technologies Corp. (Foster City, CA, USA) and used at 1:1,000 v/v dilution; the specificity of the antibody was verified based on the molecular size of the double protein band identified by immunoblotting and the staining of apically-proximal cell-cell junctions in immunofluorescence experiments. Rabbit anti-Par3 polyclonal antibody (catalogue number 07-330) was purchased from Upstate, Millipore (Millipore, CA) and used at 1:1,000 v/v dilution; the specificity of the antibody was verified based on the molecular size of the triple protein band identified by immunoblotting, its downregulation after silencing with siRNAs and the staining of apically-proximal cell-cell junctions in immunofluorescence experiments. Mouse anti-E-cadherin monoclonal antibody (catalogue number 610182) was purchased from Transduction Laboratories/Becton Dickinson AB (Stockholm, Sweden) and used at 1:20,000 v/v dilution; the specificity of the antibody was verified based on the molecular size of the single protein band identified by immunoblotting, its downregulation by TGFβ signaling and the staining of medially-proximal cell-cell junctions in immunofluorescence experiments. Rabbit anti-SIK polyclonal antibody was made in-house as reported before [34] and used at 1:500 v/v dilution; the specificity of the antibody was verified based on the molecular size of the single protein band identified by immunoblotting, its induction by TGFβ signaling and its downregulation after silencing with siRNAs. Rabbit polyclonal anti-CAR antibody was a kind gift from T. Vincent, LICR, Stockholm, Sweden, and was used at 1:500 v/v dilution; the specificity of the antibody was verified based on the molecular size of the single protein band identified by immunoblotting, its downregulation by TGFβ signaling and the staining of apically-proximal cell-cell junctions in immunofluorescence experiments. As secondary antibodies we used: goat anti-rabbit antibody conjugated to horseradish peroxidase (catalogue number 656120, lot number 1576428A), purchased from Invitrogen/Life Technologies Corp. (Foster City, CA, USA) and used at 1:40,000 v/v dilution. Goat anti-mouse antibody conjugated to horseradish peroxidase (catalogue number 626520, lot number 1629505A), purchased from Invitrogen/Life Technologies Corp. (Foster City, CA, USA) and used at 1:20,000 v/v dilution. Secondary tetramethyl-rhodamine iso-thiocyanate (TRITC)-conjugated goat anti-rat antibody was from Jackson Immunosearch Laboratories, Inc. (West Baltimore, PA) and was used at 1:5,000 v/v dilution. Secondary anti-rabbit or anti-mouse antibodies conjugated to Alexa fluor 546 and 488 were from Molecular Probes (Invitrogen, Corp., Carlsbad, CA) and were used at 1:5,000 v/v dilution.

### Adenoviral infections and transient transfections

Adenovirus expressing human SIK epitope-tagged with Flag at its N-terminus was constructed using the Adeno-X kit from BD Clontech, Mountain View, CA. Adenoviruses were amplified and titrated in HEK293 cells, as described [22]. Adenoviral infections were performed as described [22], without observing any signs of cytotoxicity, leading to a rate of 75-90% infected epithelial cells, as assessed by live GFP autofluorescence and immunofluorescence microscopy.

HEK293T cells were transiently transfected via the calcium phosphate protocol, as described [64]. NMuMG cells were transiently transfected twice, allowing 24 h between transfections, with siRNAs as previously described [22, 34].

### Immunoblotting and immunoprecipitation assays

SDS-PAGE, immunoblot and co-immunoprecipitation analysis was performed as described [22, 64]. In addition, Protein-G Dynabeads^®^ (Invitrogen, Corp., Carlsbad, CA) were used for Figure 1A according to the manufacturer’s protocol. The *in vitro* phosphorylation assay using incubation with [^32^P] ATP after Par3 immunoprecipitation (Figure 1D) was performed using cell lysates from transiently transfected NMuMG cells, as previously described [65]. The efficiency of immunoblotting and equal loading of proteins was verified by staining of the nitrocellulose membrane with 0.1% w/v Ponceau-S in acetic acid. Upon incubation with primary antibodies and horseradish peroxidase-conjugated secondary antibodies (see antibody list above), enhanced chemiluminescence assays were performed using the Millipore kit (Merck/Millipore, Billerica, MA, USA) and the immunoblots were developed at ambient temperature and during the first few minutes of the reaction that corresponded to the linear range of light emission; autoradiograms and immunoblots were exposed on X-ray films, which were scanned and quantified by the AIDA software (FujiFilm Sweden, Stockholm, Sweden) or immunoblots were analyzed in an automated imaging system (Bio-Rad Laboratories AB, Solna, Sweden) and the corresponding software Quantity One. Digital image memory content was reduced and brightness-contrast was adjusted using Adobe Photoshop CS3. Densitometric quantification of protein bands was performed using the Image J software (Image J, NIH). After subtracting the background signal the band representing the protein of interest was subsequently normalized *versus* its loading control (α-tubulin, β-tubulin or GAPDH).

### Immunofluorescence microscopy

Approximately 95% confluent infected NMuMG monolayers were analyzed by immunofluorescence 36-48 h post-infection or 48 h post-siRNA transfection as described [34]. Cells were counterstained with 4’,6’-diamidino-2-phenylindole (DAPI) to visualize the nuclei. A Zeiss Axioplan 2 immunofluorescence microscope was used with the Zeiss 20×, 40× or 63× objective lens and photographing at ambient temperature in the presence of immersion oil. Images were acquired with a Hamamatsu C4742-95 CCD digital camera and the acquisition software QED Camera Plugin v1.1.6 (QED Imaging Inc.) and Volocity^®^ (PerkinElmer). Images taken were processed with Adobe Photoshop 6.0 or CS2 to reduce file size.

### Clinical material

Samples of the cervical, rectal and breast cancers were obtained from the tissue bank of the Department of Oncology and Medical Radiology, Lviv National Medical University, Ukraine, in accordance to the permission of the local ethical committee. Tissue biopsies were obtained during the surgery prior to any other treatment. Formalin-fixed paraffin embedded biopsies were used for immunohistochemistry analysis.

Prostate cancer samples were obtained from the tissue bank of the C. Busch laboratory at Uppsala University and were collected based on methods and ethical permissions described before [66, 67].

### Immunohistochemistry

Paraffin embedded tissue samples were cut at 4 µm and after hydration, antigen exposure was obtained through boiling in citrate buffer for 5 min at 750 W, repeated twice, in a microwave. Endogenous peroxidase activity was quenched with 3% H_2_O_2_ in PBT (PBS containing 1% v/v Tween-20) for 10 min, followed by washing in PBT. Blocking was done in 20% v/v goat serum (DAKO Sweden AB, Stockholm) in PBT for 1 h. After blocking and subsequent washing in PBT, the slides were incubated with the primary antibody overnight at 4 °C in a humidified chamber. The next day, the slides were incubated with goat anti-rabbit biotin labeled secondary antibodies (DAKO Sweden AB, Stockholm) for 45 min at room temperature. After washing, HRP was coupled to the secondary antibody with the Vectastain ABC kit (Vector Laboratories, Inc., Burlingame, CA, USA). Finally, HRP substrate (DAB, Vector Laboratories, Inc., Burlingame, CA, USA) was added to the slides for 10 min, the slides were counterstained with hematoxylin for 30 sec and dehydrated before mounting with Mountex (Histolab Products AB, Västra Frölunda, Sweden). Images were captured with a Leica DM4500B (camera DFC320, ocular 10 ×, objectives 20 ×/0.50 HC PL and 40 ×, 506145) and the Leica Application Suite software, version 2.4.0 (Wetzlar, Germany) as 16-bit depth .tif format images with 48-bit image resolution, and expression of the analyzed proteins was scored manually.

The breast cancer samples used for immunohistochemistry of SIK, Par3 and phosphorylated Smad2 were also analyzed for DNA ploidy. The slides carrying the tumor tissue were stained with the Feulgen method and image cytometry was used to quantify the DNA content in single cells in the tissue [68]. DNA ploidy histograms were then built leading to DNA content classification as: genomically stable diploid characterized by a narrow stem line in the 2c region and genomically unstable diploid characterized by a broad stem line in the 2c region that expanded towards the 4c region; genomically stable aneuploid characterized by histograms with a narrow peak outside the 2c region; genomically unstable aneuploid characterized by a broad peak outside the 2c region and additional peaks exceeding the 4c region (data not shown). SIK immunohistochemistry resulted in both nuclear and cytoplasmic staining and individual tumor cells were grouped into two clusters, those with nuclear (cells exhibiting strictly nuclear or both nuclear and cytoplasmic localization) and those with cytoplasmic (cells exhibiting strictly cytoplasmic localization or undetectable expression) SIK localization. The data were statistically analyzed for correlation between nuclear SIK expression in the four DNA ploidy groups using ANOVA and the Kruskall-Wallis statistic (Supplementary Figure 4B).

### Analysis of HPA and patient survival based on clinical samples

The human protein atlas (HPA, https://www.proteinatlas.org/) was used as a source of human cancer patient immunohistochemistry data derived from cancer tissue microarrays. The on-line HPA tool generated tab-separated files including the Ensembl gene identifier (“Gene”), gene name (“Gene name”), tumor name (“Cancer”), the number of patients annotated for different staining levels of each protein (“High”, “Medium”, “Low” and “Undetectable”). All data are based on The Human Protein Atlas version 17. Immunohistochemistry for SIK was performed based on two antibodies: our home-made antibody described above [34] and the HPA038211 anti-SIK rabbit antibody (Sigma-Aldrich Sweden AB, Täby, Sweden); immunohistochemistry for Par3 was performed based on the single HPA030443 anti-Par3 antibody (Sigma-Aldrich Sweden AB, Täby, Sweden).

Using the Kaplan-Meier plotter web-tool (http://kmplot.com/analysis/) we analyzed the relationship between *SIK* mRNA expression and patient survival. The cancer patient expression and survival data are derived from the gene expression omnibus (GEO, https://www.ncbi.nlm.nih.gov/geo/), the European genome-phenome archive (EGA, https://www.ebi.ac.uk/ega/home) and the cancer genome atlas (TCGA, https://cancergenome.nih.gov/) databases. To obtain clinical outcome according to *SIK* gene expression, patients were classified into two groups, low and high, based on quantile *SIK* expression generated by the program. Based on this analysis, three tumor types were selected, breast, stomach and ovarian carcinomas. In breast carcinomas, we calculated the median post-progression survival (PPS) of the patients in all tumor grades. In stomach carcinoma, the median overall survival (OS) was calculated for patients with stage one carcinoma only. Progression free survival (PFS) of the patients with ovarian carcinoma was calculated in patients with stage IV carcinoma. Kaplan-Meier survival curves were calculated with a corresponding hazard ratio (HR) in the 95% confidence interval the statistical significance was calculated after a χ-square statistical test with probability value smaller than 0.02 indicating significance.

### Real-time RT-PCR

Total NMuMG RNA was analyzed by quantitative RT-PCR as described [22, 34], using specific PCR primers for *CAR* (forward 5’-ATCGTTTACCTGCAAGCCACG-3’ and reverse 5’-GCTCAAACCACTGGTGAAATC-3’), *ZO-1* (forward 5’-AGAGGACTTGTCAGCTCAGCC-3’ and reverse 5’-GTCTTAGGAATCCAGCTTCTCG-3’) and *SIK* (forward 5’-CCGGGTGGGCTTTTACG-3’ and reverse 5’-TTATTGCAACCTGCGTTTTGG-3’) cDNA, and *glyceraldehyde-3’-phosphate dehydrogenase* (*Gapdh*) (forward 5’-TGTGTCCGTCGTGGATCTGA-3’ and reverse 5’-CCTGCTTCACCACCTTCTTGA-3’) was used as the reference gene.

### Statistical analysis

The differences between TGFβ-inducible mRNA levels under control or SIK specific gene silencing conditions were evaluated statistically using a standard two-tailed paired Student’s t-test for samples with unequal variance. Significance is reported at *p* < 0.05. Data are plotted in bar graphs that represent average values from triplicate determinations with standard deviations. Each experiment was repeated two to five times, which represents biological repeats, and each of these included three technical repeats. Statistical analysis of Kaplan-Meier survival analysis and tumor tissue DNA ploidy analysis was explained in the corresponding method section.

## SUPPLEMENTARY MATERIALS FIGURES


