# Supplementary material for: The protein kinase SIK downregulates the polarity protein Par3
Source: Oncotarget. 2017 Dec 31;9(5):5716–35. doi: 10.18632/oncotarget.23788 (PMC5814169; doi:10.18632/oncotarget.23788)
Supplement: Supplementary file 1 [file oncotarget-09-5716-s001.pdf]

The protein kinase SIK downregulates the polarity protein Par3

SUPPLEMENTARY MATERIALS

	Sequence	Species
PAR-3	SPSR----DVGPSLGLKKSSSLESLQTAVA	*Homo sapiens*
	SPSR----DVGPSLGLKKSSSLESLQTAVA	*Mus musculus*
	SPNR----DVGPSLGLKKSSSLESLQTAVA	*Rattus norvegicus*
	SPTR----DVGPSLGLKKSSSLESLQTAVA	*Gallus gallus*
	SSTR----DVGPSLGLKKSSSLESLQTAVA	*Danio rerio*
	SPSR----DVGPSLGLKKSSSLESLQTAVA	*Xenopus laevis*
	SPTR----DVGPSLGLKKSSSLESLQTAVA	*Anolis carolinensis*
	Q--------LGPSLGMKKSSSLESLQTMVQ	*Tribolium castaneum*
	PPKQ----QLGPSLGLKKSSSLESLQTMVQ	*Acyrthosiphon pisum*
	SSSK----TMGPGLGMEKSSSLESLQVAVA	*Strongylocentrotus purpuratus*
	AEARDQLGDLGPSLGMKKSSSLESLQTMVQ	*Drosophila melanogaster*
	ASTNSQNLDDSDMLN-RRSQSMESI-----	*Caenorhabditis elegans*

SIK-Motif ...I.KS.**S**...I...
L RT L
V V

Supplementary Figure 1: Sequence alignment of the predicted phosphorylation site across species. The predicted phosphorylation site for SIK in Par3 is aligned with its orthologs in selected species. The phosphorylated serine is indicated in black, while the conserved amino acids are depicted in green. A consensus phosphorylation motif is shown at the bottom.

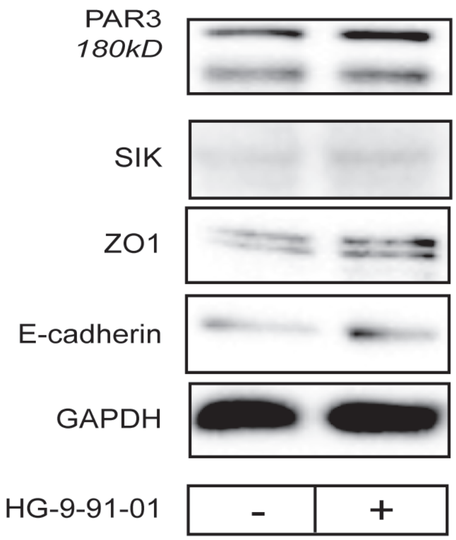

Supplementary Figure 2: The SIK inhibitor HG-9-91-01 stabilizes Par3, AJ and TJ proteins. NMuMG cells were treated with HG-9-91-01 (1 µM) and endogenous levels of Par3, SIK, TJ protein ZO-1 and AJ protein E-cadherin, were assessed by immunoblotting. GAPDH was used as a loading control. Representative immunoblot out of at least two repeats is shown. Molecular size markers in kilo Dalton (kD) are also marked.

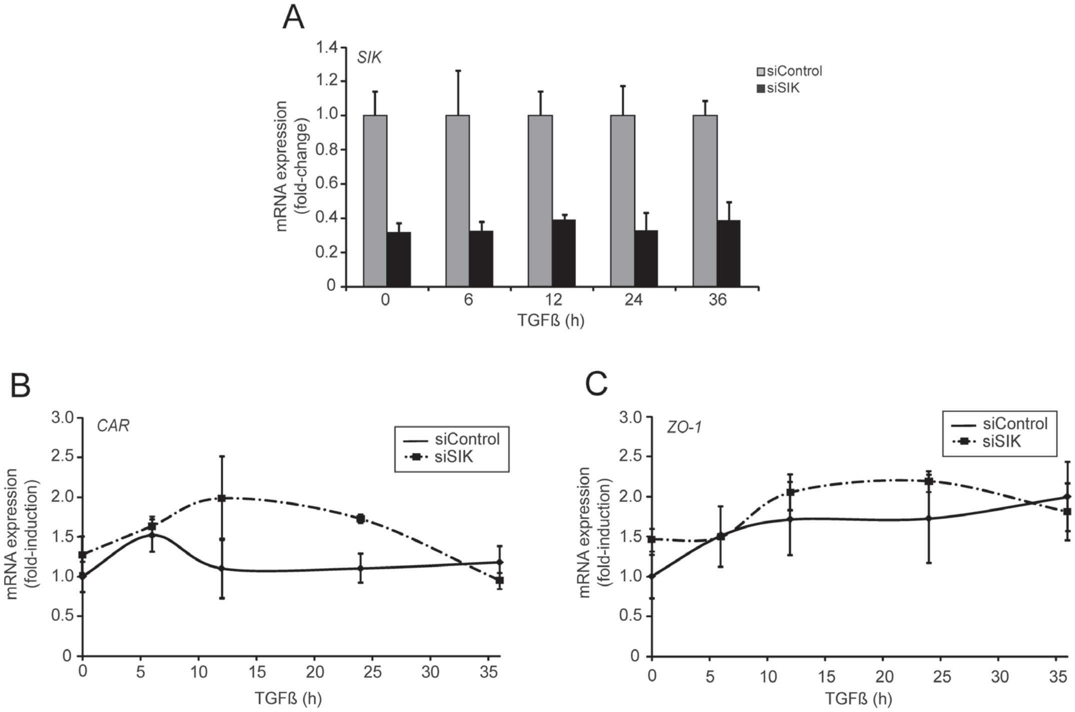

Supplementary Figure 3: SIK downregulation does not affect transcription of TJ proteins. **A**. Verification of knockdown levels of *SIK* in this experiment, representative for knockdowns in the other figures. In all panels, the specific mRNA levels were normalized to the levels of the housekeeping gene *Gapdh*. **B, C**. NMuMG cells were transfected with siControl or siSIK 24 h prior and at the moment of stimulation with TGFβ (5 ng/ml) for 0, 6, 12, 24 and 36 h. Relative mRNA levels for *CAR* (B) and *ZO-1* (**C**) do not change significantly. Average values from triplicate determinations and the corresponding standard deviations are graphed. The figure shows representative experiments from two or more repeats.

A

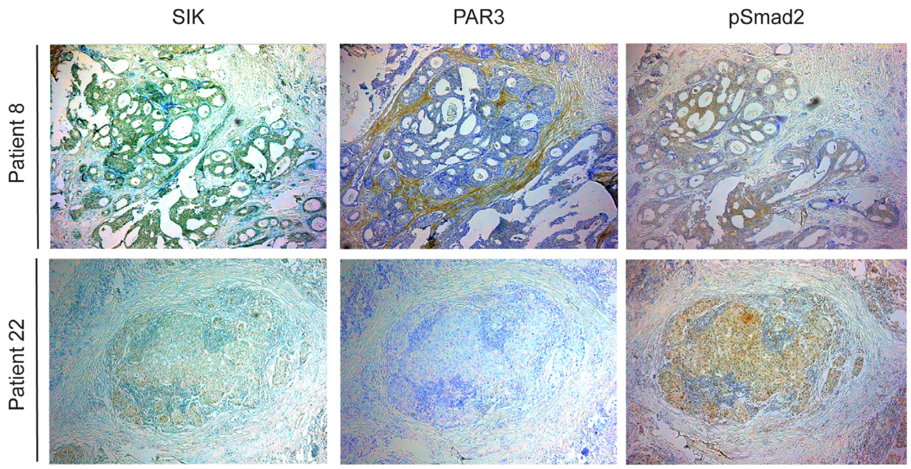

B

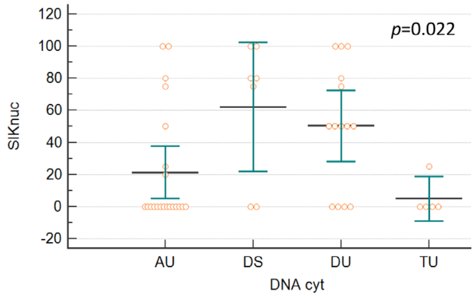

Kruskal-Wallis test

Test statistic	8,3031
Corrected for ties Ht	9,6448
Degrees of Freedom (DF)	3
Significance level	P = 0,021840

Post-hoc analysis

Factor	n	Average Rank	Different (P<0,05) from factor nr
(1) AU	21	19,93	(2)(3)
(2) DS	7	32,14	(1)(4)
(3) DU	14	29,14	(1)(4)
(4) TU	5	15,30	(2)(3)

Supplementary Figure 4: SIK and cancer association. **A**. Comparison of expression of SIK, Par3 and phospho-Smad2 (pSmad2) in two patients with ductal breast carcinoma, with the lower panel showing serial sections of the same tumor specimen. **B**. Comparison between expression of nuclear SIK protein in human ductal breast carcinoma evaluated by immunohistochemistry. The % of nuclei exhibiting nuclear SIK (SIKnuc) in human ductal breast carcinoma tissue immunostained for SIK is plotted relative to the degree of ploidy of the tumor cells, verified based on DNA cytology (DNA cyt). Tumor cells exhibiting both nuclear and cytoplasmic SIK were scored as positive for nuclear SIK expression; tumor cells with only cytoplasmic SIK or undetectable SIK staining were scored as negative for nuclear SIK. The ploidy of tumor cells is classified as aneuploidy unstable (AU, n=21), diploid stable (DS, n=7), diploid unstable (DU, n=14) and tetraploid unstable (TU, n=5). Data points are plotted as circles with horizontal lines indicating statistically significant differences between nuclear SIK expression in the four groups under comparison (ANOVA, Kruskall–Wallis, $p=0.01$).
